# Explaining *Ayurvedic* preparation of *Rasasindura*, its toxicological effects on NIH3T3 cell line and zebrafish larvae

**DOI:** 10.1016/j.jaim.2021.08.011

**Published:** 2021-11-29

**Authors:** Snehasis Biswas, Jayesh Bellare

**Affiliations:** a Department of Chemical Engineering, Indian Institute of Technology, Powai, Mumbai, 400076, India; b Wadhwani Research Centre for Bioengineering, Indian Institute of Technology, Powai, Mumbai, 400076, India

**Keywords:** *Rasasindura*, *Kajjali*, Cell culture, Zebrafish larvae, *Ayurveda*

## Abstract

*Rasasindura* is a mercury-based medicinal formulation that contains HgS (>99%). Although cinnabar ore was a well-known mineral in the past, the *Ayurvedic* practitioner adopted a critical and tedious procedure for the preparation of *Rasasindura*. Therefore, it is essential to understand the *Ayurvedic* process in the perspective of material science. Further, a toxicity study is also required as mercury is the main component in *Rasasindura*. Here, in the present study, we characterized *Rasasindura* and one of its intermediates (*Kajjali*) to understand the physicochemical changes that occur in the *Ayurvedic* process. Furthermore, we have assessed the toxicity of *Kajjali* and *Rasasindura* in NIH3T3 cell lines and zebrafish larvae. XRD analysis of *Rasasindura* confirms it as a highly pure α-HgS with size ranges from nano to micron sizes (starting from ∼80 nm). Whereas, *Kajjali* is a β-HgS having lower size ranges (starting from ∼30 nm). *Rasasindura* did not show significant cytotoxicity on NIH3T3 cell line up to 75 ppm, whereas for *Kajjali*, cytotoxicity was observed above 20 ppm. The higher toxicity of *Kajjali* is due to higher penetration of particles into the cells. However, in zebrafish larvae, even at too high concentrations (1000 ppm), both *Rasasindura* and *Kajjali* did not show any toxicity or morphological changes. This study concludes that *Rasasindura* is not toxic up to a reasonable concentration. Further, these two drugs did not contain toxic organic mercuric compound; otherwise, it could have been lethal to the zebrafish larvae.

## 1 Introduction

*Ayurveda* is one of the oldest medicinal systems in human society which had originated more than 5000 years ago [1]. Interestingly, *Ayurveda* uses metal-based medicine in therapeutic applications. Numerous *Ayurvedic* medicines contain metals (or metal compounds) such as gold, silver, lead and arsenic, which are not essential elements for humans. The most astonishing fact is that mercury, which is a heavily toxic metal, is one of the most common ingredients in *Ayurvedic* medicines. Although WHO has advocated to restrict the use of mercury in medicinal applications, *Ayurveda* still uses these mercury-based medicines for multiple therapeutic purposes.

The importance of mercury in *Ayurveda* can be understood by the fact that a sub-branch of *Ayurveda*, *Rasa Shastra*, was named after mercury (in *Sanskrit*, *Rasa* means mercury) [2]. *Rasasindura* is a mercury-based medicine that is used to treat high fever, jaundice, sexual diseases, immune and nervous system related diseases [3]. *Kajjali* is another important medicine in *Ayurveda* which is an intermediate product in the *Ayurvedic* preparation process of *Rasasindura* and this is used as a rejuvenating agent [4]. Ayurvedic process of Rasasindura manufacturing is tedious and requires several days for preparing the final product as it involves several steps such as purification, mixing heat-treating steps. The raw materials required for the manufacturing process are liquid mercury and solid sulphur. Several physical and chemical transformations occur during the Rasasindura preparation. Therefore, the first objective of this study is to understand the *Ayurvedic* manufacturing process starting from raw mercury and sulphur. Moreover, various organic juices are used in the manufacturing process that could lead to the presence of organic mercury in *Kajjali* and *Rasasindura*. As organic mercury compounds such as methyl mercury are extremely toxic to the biological system, a small amount of them could lead to a severe adverse effects in patients. Furthermore, the presence of free mercury is also a concern. Therefore, to observe the toxicological effects, we have used NIH3T3 cell line and zebrafish larvae. We used very high concentrations of *Rasasindura* and *Kajjali* (up to 1000 ppm) and examined the alteration of various parameters such as viability, reactive oxidative species (ROS), particle uptake and morphology.

## 2 Materials and methods

### 2.1 Chemicals

*Ayurvedic Kajjali* and *Rasasindura* were gifted for research purpose by Shree Dhootapapeshwar Limited, Mumbai, India. The 2′,7′-dichlorodihydrofluorescein diacetate (DCFDA) and 3-(4,5-dimethylthiazol-2-yl)-2,5-diphenyl tetrazolium bromide (MTT) were purchased from Sigma Aldrich. All chemicals for cell culture experiment was procured from Himedia, India.

### 2.2 Preparation of Rasasindura

The Ayurvedic preparation of Rasasindura has been described in our previous work [5]. A flow chart of the preparation steps has been included in the supplementary file (Fig. S1).

### 2.3 Physicochemical characterization

#### 2.3.1 Crystallographic identification

The crystal phase identification of all samples was carried out using X-ray diffraction (XRD, SmartLab, Rigaku, Japan). XRD peaks were matched with the ICDD (International Centre for Diffraction Data) database. High-Temperature XRD (HTXRD) was carried out for Kajjali to understand the crystallographic changes occurring in the Rasasindura preparation procedure. The scanning was done at various temperatures between 25 °C and 325 °C in the air environment.

#### 2.3.2 Particle size analysis

The particle size of *Kajjali* and *Rasasindura* was analyzed by dynamic light scattering (DLS) and transmission electron microscopy (TEM). For the DLS study, Malvern ZEN 1600 (Malvern Panalytical Ltd, United Kingdom) was used. For the DLS analysis, *Kajjali* and *Rasasindura* were suspended in isopropanol (1 mg/ml) and sonicated for 10 min prior to the analysis. TEM study was carried using a JEOL 2100 (JEOL, Japan) microscope operated at 200 kV. For the TEM sample preparation, suspension of particles was made similar to the DLS technique. The suspended particles were placed on a carbon-coated copper grid and dried before analysis.

#### 2.3.3 Morphological and elemental analysis

The morphology of *Kajjali* and *Rasasindura* particles were analyzed using scanning electron microscopy (SEM, JEOL, Japan). For the elemental quantification, Energy-dispersive X-ray spectroscopy, (EDAX, Oxford instrument) was employed, which was attached with scanning electron microscopy (SEM).

#### 2.3.4 Thermogravimetric analysis

Thermal analysis was carried out with thermogravimetric analysis (TGA) attached with differential thermal analysis (DTA). The DTA-TGA (Perkin Elmer, USA) experiment was conducted in an air atmosphere. The rate of temperature increase was 10 °C/min.

#### 2.3.5 XPS

The surface elemental analysis was conducted by X-ray Photoelectron Spectroscopy (XPS, Kratos Analytical, Japan) equipped with a monochromatic X-ray source of 1486.6 eV. The peak position (binding energies) was calibrated with a standard gold peak (Au 4f7/2) at a position of 83.95 eV. The XPS peaks were analyzed and de-convoluted with the help of *ESCApe*^*TM*^ software, Kratos Analytical.

### 2.4 Exposure of Kajjali and *Rasasindura* to NIH3T3 cell line

The NIH3T3 (mouse fibroblast) cell line was obtained from National Centre for Cell Science, Pune, India. NIH3T3 cell line was cultured in Dulbecco's modified Eagle medium (DMEM) having 10% fetal bovine serum (FBS), 1% l-glutamine and 0.1% antibiotic. The cells were incubated in 5% CO_2_ atmosphere at 37 °C. *Kajjali* and *Rasasindura* particles were suspended by sonicating it in the cell culture media for 10 min. The suspended *Kajjali* and *Rasasindura* particles were exposed to the adherent NIH3T3 cell line at various concentrations from 10 to 1000 ppm. For cell viability assessment, MTT [3-(4,5-dimethylthiazol-2-yl)-2,5-diphenyl tetrazolium bromide] assay was conducted in 96-well plate. Reactive oxygen species (ROS) was also studied in the black bottom 96-well plate by 2′,7′-dichlorodihydrofluorescein diacetate (DCFDA) method. Cell viability tests and ROS experiments were performed at various time points after drug exposure. For cell morphology study, the images were captured by confocal microscopy, followed by FITC and PI staining after 48 h from drug exposure. The fluorescence-activated cell sorting (FACS) flow cytometry study was conducted (48 h after treatment) after propidium iodide (PI) staining to confirm particles uptake by cells. SEM imaging of drug-treated cells was also conducted.

### 2.5 Exposure of Kajjali and *Rasasindura* to zebrafish larvae

The zebrafish experiment was done as per The Committee for the Purpose of Control and Supervision of Experiments on Animals (CPCSEA), India. Adult zebrafish were maintained as per our previous studies [6,7]. Embryos were obtained after mating of adult male and female fishes. The embryos were maintained in E3 medium. Larvae were kept in reverse osmosis-filtered water (pH 6.5–7.5), whose salinity was reconstituted to ∼500 μS. The zebrafish larvae (4 dayspost-fertilization, dpf) were exposed to various concentrations of *Kajjali* and *Rasasindura*. The *Kajjali* -*Rasasindura* water suspension was sonicated for 10 min and exposed to transparent zebrafish larvae to check the morphological and ROS changes. The images were captured using a stereomicroscope (SZX7, Olympus, Japan).

ROS of zebrafish larvae was carried out by the whole-mount method using DCFDA fluorescent probe [8]. For whole-mounted ROS detection, after 48 h of *Rasasindura* exposure, the larvae were anaesthetized using 0.05 mg/ml tricaine (MS-222) solution. After 10 min, the larvae were washed using PBS buffer (pH 7.4) twice and incubated with DCFDA for 30 min in 28 °C. The DCFDA labelled larvae were mounted on methylcellulose and photographed under a stereoscopic microscope.

### 2.6 Statistical analysis

Statistical analysis was carried out using one-way ANOVA technique in Origin-2018 (OriginLab) software. For statistical significance, Tukey's post hoc test was carried out with ∗p < 0.05, ∗∗p < 0.001 and ∗∗∗p < 0.0001 v/s controls.

## 3 Results and discussion

### 3.1 Understanding the *Rasasindura* preparation process

In this section, the *Ayurvedic* process has been observed and documented in terms of changes in the physicochemical properties of the materials involved during the *Rasasindura* preparation. The first step of *Rasasindura* preparation is mixing of purified solid sulphur (S) and purified liquid mercury (Hg) in a ball mill for 36 h, which yields *Kajjali* (black powder).

**Fig. 1:**
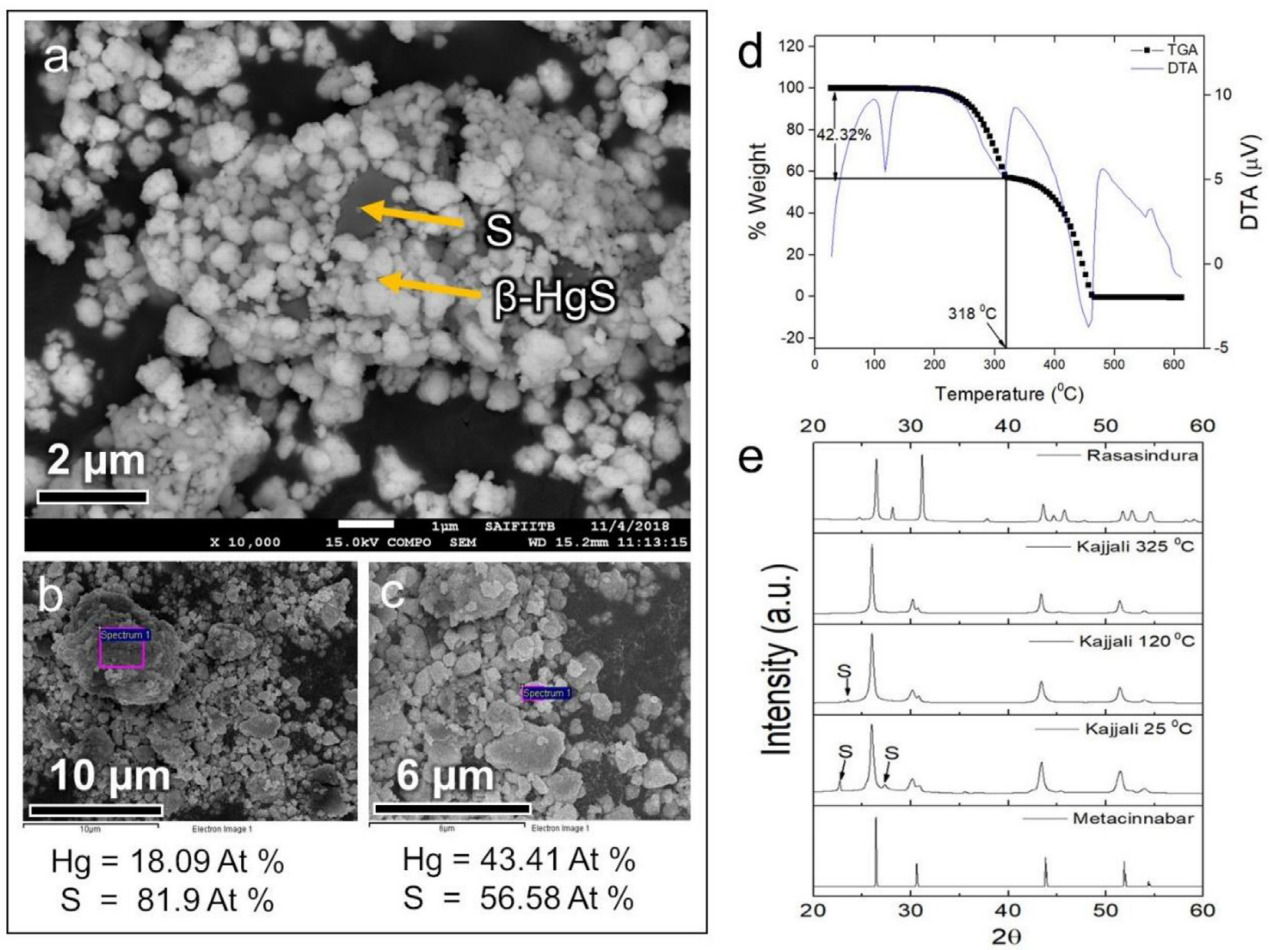
Conversion of *Kajjali* to *Rasasindura*, a) SEM backscatter image of *Kajjali*, b) and c) selected area EDAX results on big and small particles in *Kajjali*, d) DTA-TGA of *Kajjali* e) High-temperature XRD of *Kajjali* (at 25 °C, 120 °C and 325 °C) with comparison to metacinnabar and *Rasasindura*.

EDAX results of *Kajjali* at different regions showed that concentration of small size mercury particles was higher than that of large particles (Fig. 1c). Also, Hg to S ratio (Hg:S) for smaller particles was found to be 43.41:56.58, which is closer to the original stoichiometric composition of HgS (50:50). At the same time, large particles had a ratio of (Hg:S) was 18.09:81.90 (Fig. 1b). These observations can be explained as follows: in the mixing step, when liquid mercury was mechanically mixed with solid sulphur for 36 h, the HgS phase formed on the surface of sulphur particles (Fig. 1a) and the Hg converted to HgS completely having a wide range of particle sizes. The excess sulphur that remained combined with larger Hg particles.

In the next step, *Kajjali* was further heated in the glass vial with controlled temperature to obtain *Rasasindura*. The loss in mass of *Kajjali* with temperature were assessed with TGA-DTA. In the heating process, the excess sulphur (42% excess) was burned down at approximately 318 °C (Fig. 1d). After complete combustion of excess sulphur, the evaporation of *Kajjali* started (∼350 °C) and it was completely decomposed at around 460 °C. But in the actual *Ayurvedic* process, after the completion of combustion of excess sulphur, the glass vial was sealed with a cap to restrict the evaporation of *Kajjali*. Without this step, the conversion of *Kajjali* (metacinnabar or β-HgS) to *Rasasindura* (α-HgS) could not be completed. This is evident in the HTXRD peaks of *Kajjali* (Fig. 1e). As the HTXRD of *Kajjali* was conducted on the open surface (on a glass plate in XRD instrument), it is inferred that *Kajjali* did not transform to *Rasasindura* up to 325 °C (Fig. 1e); and only above 350 °C, *Kajjali* started to evaporate. Since the glass vial was sealed in the *Ayurvedic* process, the evaporated *Kajjali* got sublimed at the neck of the glass bottle along with the phase transition from β-HgS to α-HgS. The sublimation is one of the reasons for the high purity of *Rasasindura*.

### 3.2 Physicochemical analysis of *Kajjali* and *Rasasindura*

**Fig. 2:**
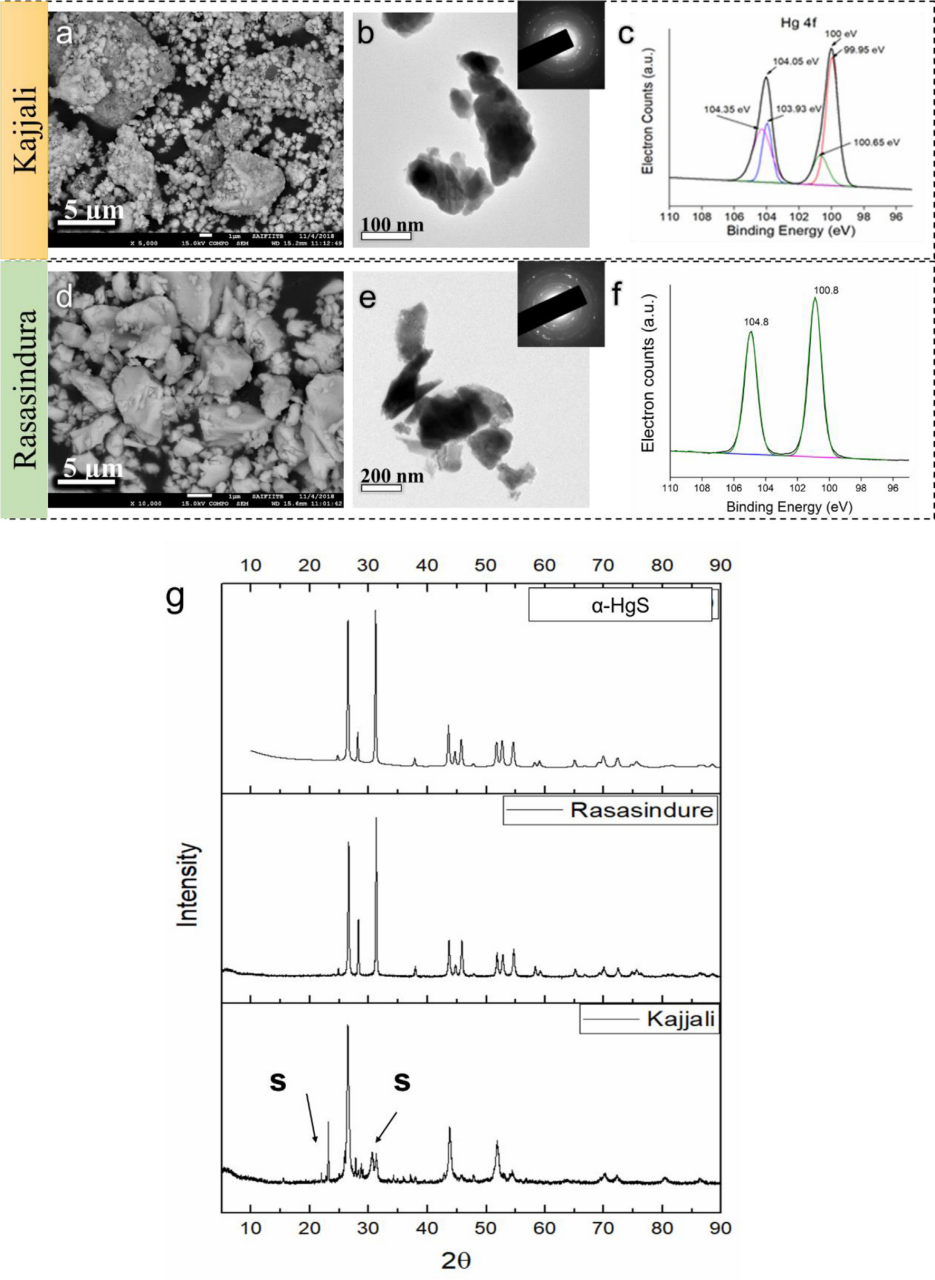
Physicochemical characterization of *Kajjali* and *Rasasindura*. a) SEM of *Kajjali*, b) TEM of *Kajjali*, c) XPS of Hg 4f region of *Kajjali*, d) SEM of *Rasasindura*, e) TEM of *Rasasindura*, and f) XPS of Hg 4f region of *Rasasindura* and g) XRD of *Kajjali* and *Rasasindura*.

SEM study (Fig. 2a) showed the particle size distribution of *Kajjali* powder. From the TEM (Fig. 2b) studies, it was confirmed that *Kajjali* had some nano-sized particles below 100 nm. The DLS study also found particles having nano sizes (supplementary file, Fig. S2). The high-resolution XPS (HRXPS) peaks (Fig. 2c) were found at 100 eV and 104.05 eV for Hg (Hg 4f peaks) having 4.05 eV 4f_7/2_ - 4f_5/2_ splitting. After deconvolution of the XPS 4f_7/2_ peaks, two peaks were obtained at 99.95 eV and at 100.65 eV, which are close to the peak position of β-cinnabar and α-cinnabar respectively [[9], [10], [11]] and no peaks of free Hg (Hg^0^ < 99.8 eV) were obtained. The XRD (Fig. 2g) of *Kajjali* illustrated that major phases contained in *Kajjali* were the β-cinnabar (52.32 wt.%), orthorhombic sulphur (44.32 wt.%) and 3.32 wt. % α-cinnabar.

*Rasasindura* also contained nano-sized particles (<100 nm) which was confirmed by TEM (Fig. 2e) and DLS study (supplementary file, Fig. S3). From SEM images (Fig. 2d), it was observed that *Rasasindura* also contained large agglomerate particles (>1 μm). The agglomeration had been caused by the prolonged heat treatment of *Kajjali*. The XRD (Fig. 2g) profile of *Rasasindura* matched exactly with α-HgS. Therefore, it can be inferred that *Rasasindura* has a single crystalline α-HgS phase. The EDAX study at a different position (small and large particles) showed approximately similar Hg to S ratio (Hg:S = ∼84:16 wt %), which was close to the stoichiometric concentration of HgS (Hg:S = 86.22:13.78 wt.%). HRXPS profile of *Rasasindura* (Fig. 2f) at Hg 4f region showed only one 4f_7/2_ peak (after de-convolution). The 4f_7/2_ XPS peak was obtained at 100.8 eV, which closely matched with α-HgS.

### 3.3 Cell viability and ROS study

**Fig. 3:**
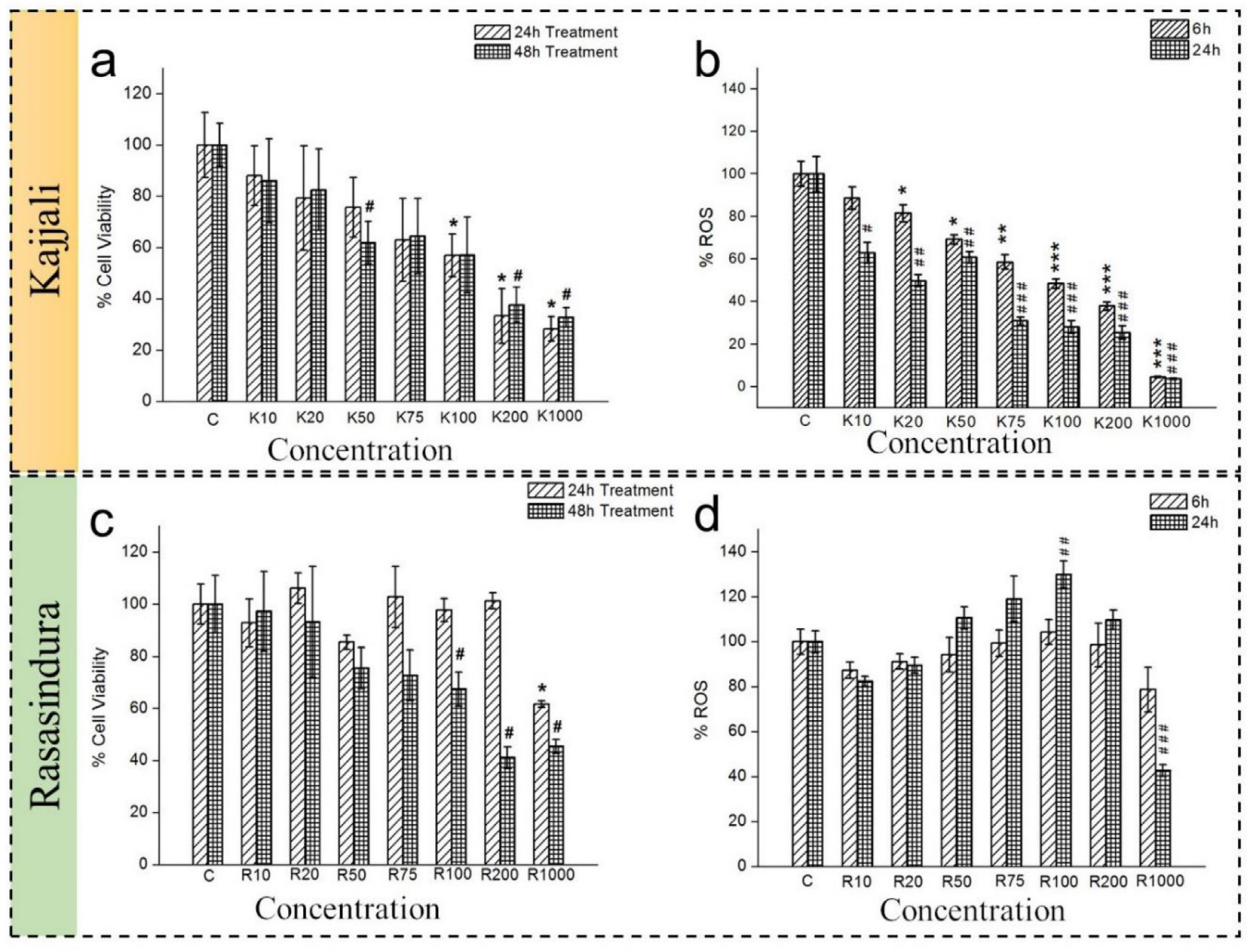
MTT assay and ROS assay of NIH3T3 cell line after (a and c) *Kajjali* and (b and d) *Rasasindura* exposure. K10 indicates 10 ppm *Kajjali*, K20 indicates 20 ppm *Kajjali* and so on. Similarly, R10 equals to 10 ppm *Rasasindura* and so on. Values are expressed as mean ± SEM with ∗p < 0.05, ∗∗p < 0.001 and ∗∗∗p < 0.0001 v/s control.

To understand the biological effects of *Kajjali* and *Rasasindura*, cell culture study was carried out using NIH3T3 cell line. The cell viability was examined using the MTT assay (Fig. 3a). The cells were treated by increasing the *Kajjali*/*Rasasindura* concentration from 10 to 1000 ppm. The cell viability was examined after 24 h and 48 h of drug exposure. At 50 ppm and concentrations above that, *Kajjali* showed significant cytotoxicity after 48 h (Fig. 3a). The ROS of *Kajjali* treated cells were found to be decreased with increasing concentration (Fig. 3b) due to cell death. The ROS results indicating ROS-independent cell death due to Kajjali exposure. Our ROS measurement time was 6 h and 24 h after Kajjali exposure. At the 6 h time point, we did not observe decreasing ROS with increasing concentration of Kajjali. It may happen due to cell death. The cell death could happen due to immediate ROS generation after Kajjali exposure (ROS may increase within the first few hours after exposure, hence it was not reflected in the 6 h ROS data). The confocal study further confirmed cell death due to *Kajjali* exposure (Supplementary file, Fig. S4).

**Fig. 4:**
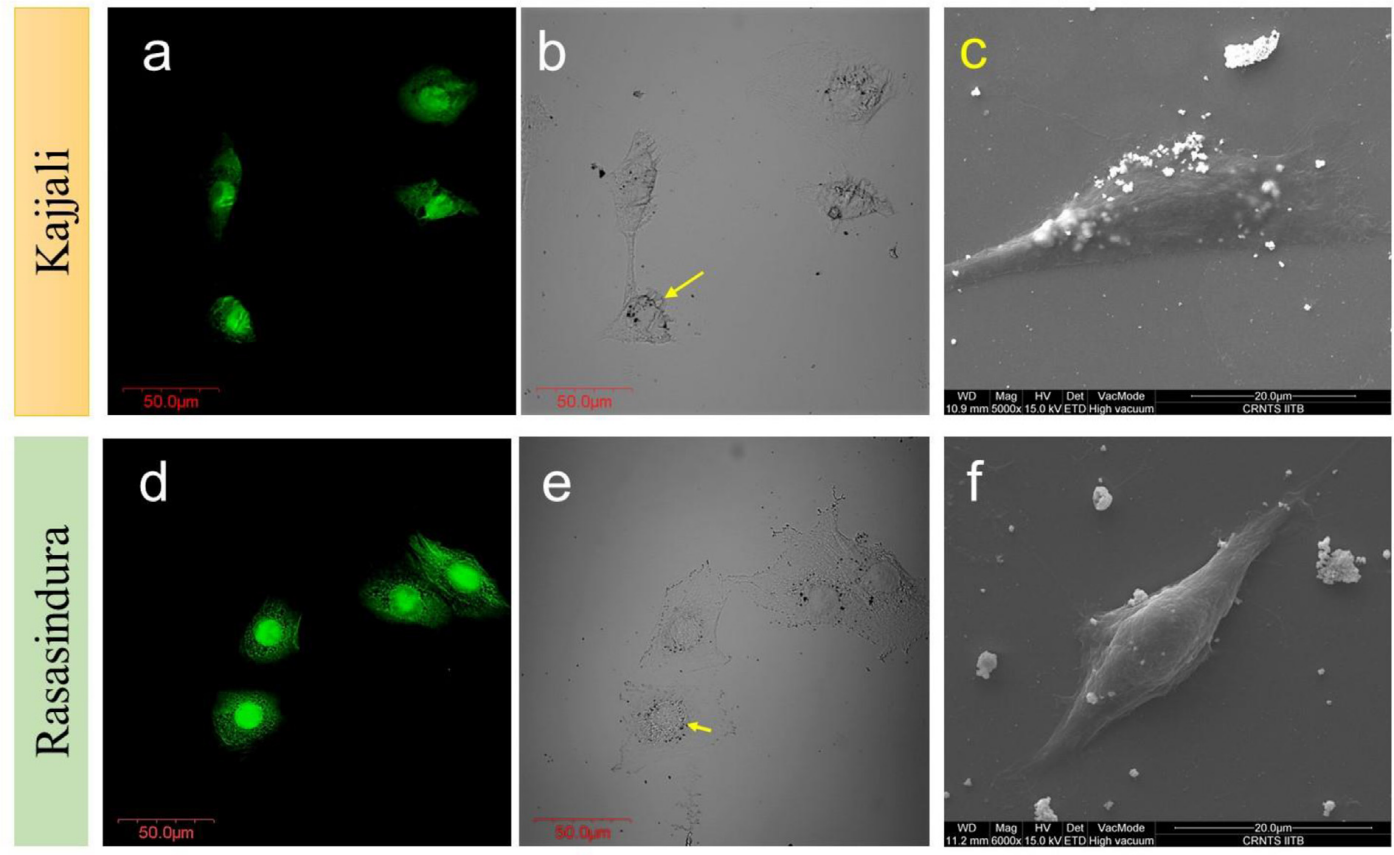
High-resolution confocal image (a, b, d and e) and SEM (c and f) images of *Kajjali* and *Rasasindura* treated cells (50 ppm) after 48 h.

On the other hand, it was observed that even at a very high concentration (up to 200 ppm), the *Rasasindura* was not cytotoxic after 24 h. However, at 48 h, 100 ppm and concentrations above that showed a significant reduction of cell viability for Rasasindura (Fig. 3c). The ROS study (Fig. 3d) at 6 h and 24 h showed that at 6 h, ROS change was insignificant up to 200 ppm concentration. However, after 24 h treatment, ROS increased as compared to the control for most of the concentrations (except 1000 ppm), but the significant variance was found at 100 ppm.

### 3.4 Cell morphology by confocal and SEM studies

The high-resolution confocal and SEM images revealed that after *Kajjali* treatment to the NIH3T3 cell line, the deformation was clearly seen in Fig. 4a–c. Deformation of the nucleus was also observed after Kajjali exposure (50 ppm).

**Fig. 5:**
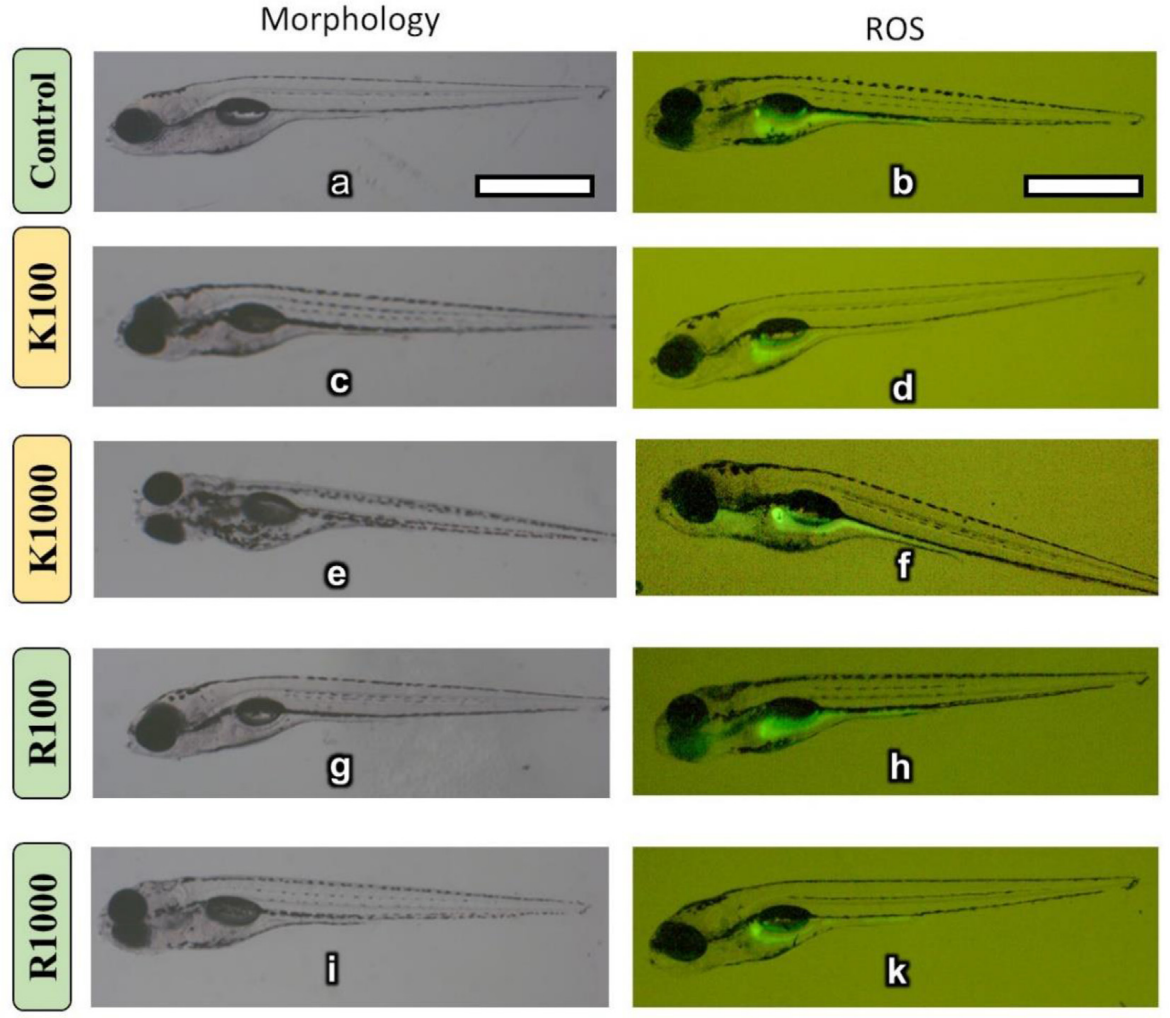
Morphology (right-sided images) and ROS (left-sided images) of *Kajjali* and *Rasasindura* treated zebrafish larvae (K100 = 100 ppm and, K1000 = 1000 ppm *Kajjali*: R100 = 100 ppm, R1000 = 1000 ppm *Rasasindura*). Treatment was done at 4 dpf and images were taken after 48 h of treatment (at 6 dpf). Scale bar = 1 mm.

On the other hand, *Rasasindura* particles were accumulated in the cells. The distribution of *Rasasindura* particles was not homogeneous among cells, as *Rasasindura* comprises a wide distribution of particle sizes. The confocal images demonstrated that particles were adsorbed on the cell membrane. The larger and agglomerated RS particles could not enter through the cell membrane; on the other hand, the smaller RS particles entered the intercellular cytosol and concentrated around the nucleus (Fig. 4e). However, the particles did not seem to enter the nucleus as no deformation was observed in the nucleus' shape (Fig. 4d–f). A considerable portion of *Rasasindura* particles accumulated in the endomembrane system surrounding the nucleus. The internalization of *Rasasindura* particles (smaller size) could occur via endocytosis [12].

### 3.5 Nanoparticle uptake measured by side scatter (SSC) vs forward scatter (FSC) signal

The cellular uptake of *Rasasindura* was further supported by the FACS study (Supplementary file, Fig. S5). It was observed from the SSC v/s FSC plot that the SSC signal increases as the concentration of *Kajjali/Rasasindura* increases [13].

### 3.6 Zebrafish larvae study

The effects of *Kajjali* and *Rasasindura* exposure on zebrafish larvae (4 dpf) at various concentrations were studied for morphological changes and ROS generation (Fig. 5). The morphology of *Kajjali* and *Rasasindura* treated larvae were observed under a stereomicroscope and no significant difference was observed. Further, from the ROS study, it was observed that there were no significant changes in the reactive oxygen species between control and intervention groups. Therefore, it can be inferred that both *Kajjali* and *Rasasindura* were non-toxic to zebrafish larvae after 48 hours of treatment. This observation suggestes that there was no (or less minimally) soluble mercury (or organic mercury) released from *Kajjali* and *Rasasindura* to the larvae medium (water) that could induce toxicity to the larvae.

From the above results, it can be summarised that the interactions of *Rasasindura* with the biological system was not destructive in both cells (up to 75 ppm) and zebrafish larvae (up to 1000 ppm). Further, cell culture studies showed that *Kajjali* was not cytotoxic up to a reasonable concentration (20 ppm). In zebrafish larval study, both *Kajjali* and *Rasasindura* did not exert any toxicity up to 1000 ppm. In our previous study [6] on adult zebrafish, no toxic effect was observed up to 70 mg/kg dose for *Rasasindura* and *Kajjali*. Moreover, in some recent biological studies, *Kajjali* and *Rasasindura* showed some beneficial effects [14,15]. However, the concerns regarding the use of mercury cannot be ignored as it is a potential neurotoxin even at lower doses. However, some recent studies have showed that HgS (*Kajjali* and *Rasasindura* mainly contain HgS) is less toxic as compared to other Hg compounds such as methylmercury, HgCl_2_ or diethyl mercury [16]. It is presumed that the low solubility of HgS could be the reason for its non-toxicity [5]. However, several mechanisms in the biological system can increase the solubility of HgS. The solubility may change due to the interaction with various enzymes which can lead to toxicity in organisms. Therefore, in the present study, the interaction of HgS with the biological system was shown that exhibits the non-toxic nature of *Rasasindura*. *Kajjali* shows higher toxicity in the cell line due to its smaller particle size as compared to *Rasasindura*. As *Kajjali* contains a higher portion of nano-sized particles, the penetration capacity of *Kajjali* particles in the cell and nucleus is far higher compared to *Rasasindura*, which makes it more toxic in the cell line study.

## 4 Conclusion

This study provides a detailed methodological insight into the *Rasasindura* preparation process. We have explained the transformation of *Rasasindura* from raw mercury and sulphur. Further, the physicochemical study of *Kajjali* and *Rasasindura* revealed their morphology, size distribution, crystallographic and elemental analysis. In summary, both *Kajjali* (up to 20 ppm after 48 h of exposure) and *Rasasindura* (up to 75 ppm after 48 h of exposure) were not found to be as toxic as compared to other Hg compounds (such as HgCl_2_, methylmercury or Hg^0^) reported in the literature. *Kajjali* and *Rasasindura* medicines, if prepared as per the *Ayurvedic* method, reduce the chances of toxicity as they do not possess any organic or soluble mercury counterparts.

## Conflict of interest

None.

## Author contributions

S.B. designed the study, collected experimental data, wrote the data, analyzed the data, and prepared the figures, and wrote the manuscript. J.B. designed the study, reviewed the manuscript, and supervision. All authors reviewed the manuscript.
