# Supplementary material for: Explaining Ayurvedic preparation of Rasasindura, its toxicological effects on NIH3T3 cell line and zebrafish larvae
Source: J Ayurveda Integr Med. 2021 Nov 29;13(2):100518. doi: 10.1016/j.jaim.2021.08.011 (PMC8728081; doi:10.1016/j.jaim.2021.08.011)
Supplement: Multimedia component 1 [file mmc1.docx]

**Explaining *Ayurvedic* preparation of *Rasasindura*, its toxicological effects on NIH3T3 cell line and zebrafish larvae**

Snehasis Biswas^1^, Jayesh Bellare^1,2^*

^1^Department of Chemical Engineering, Indian Institute of Technology, Powai, Mumbai –

400076, India

^2^Wadhwani Research Centre for Bioengineering, Indian Institute of Technology, Powai, Mumbai – 400076, India


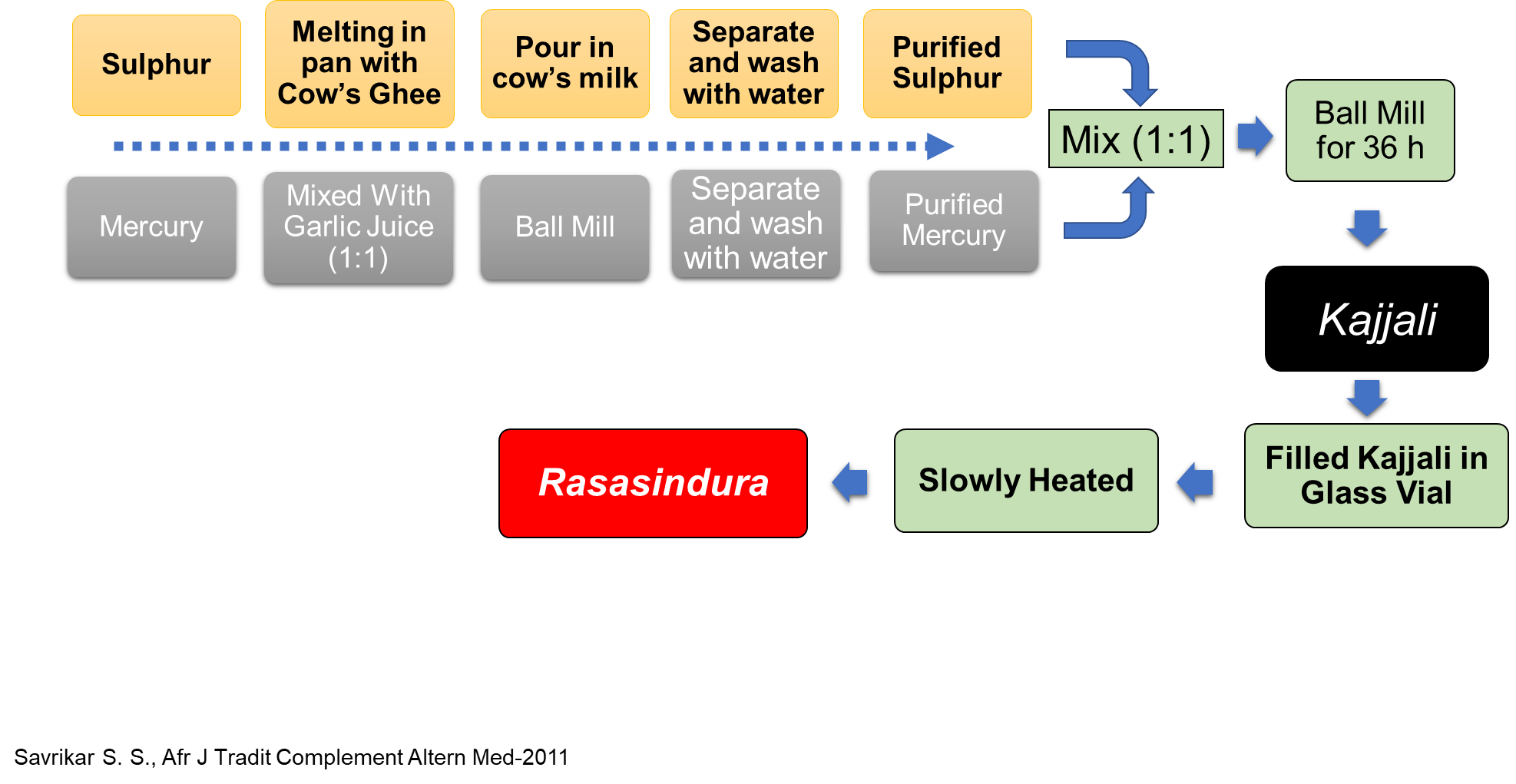


**Figure S1:** Preparation of Rasasindura by Ayurvedic method


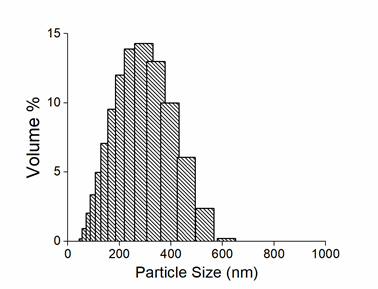


**Figure S2:** Particle size distribution of suspended particle of Kajjali (DLS study)


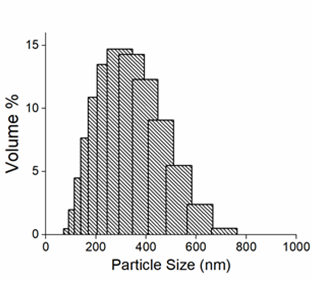

**Figure S3:** Particle size distribution of suspended particle of Rasasindura (DLS study)


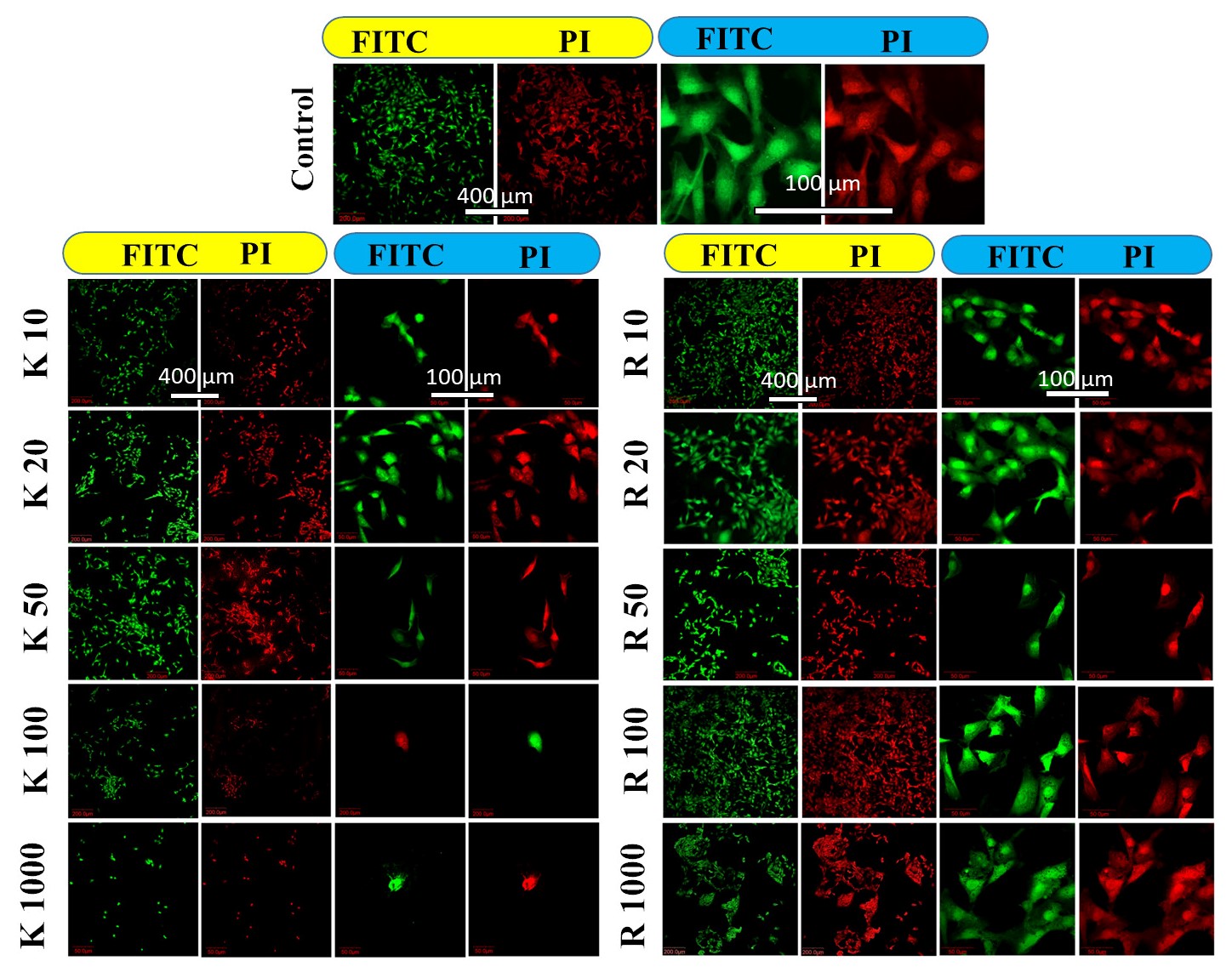


**Figure S4.** Confocal image of *Kajjali* and *Rasasindura* treated NIH3T3 cells after Fluorescein isothiocyanate (FITC) and Propidium iodide (PI) staining. K indicated *Kajjali*, R indicates Rasasindura exposure. The cell was treated at different concentrations of *Kajjali/Rasasindura,* from 10 to 1000 ppm.


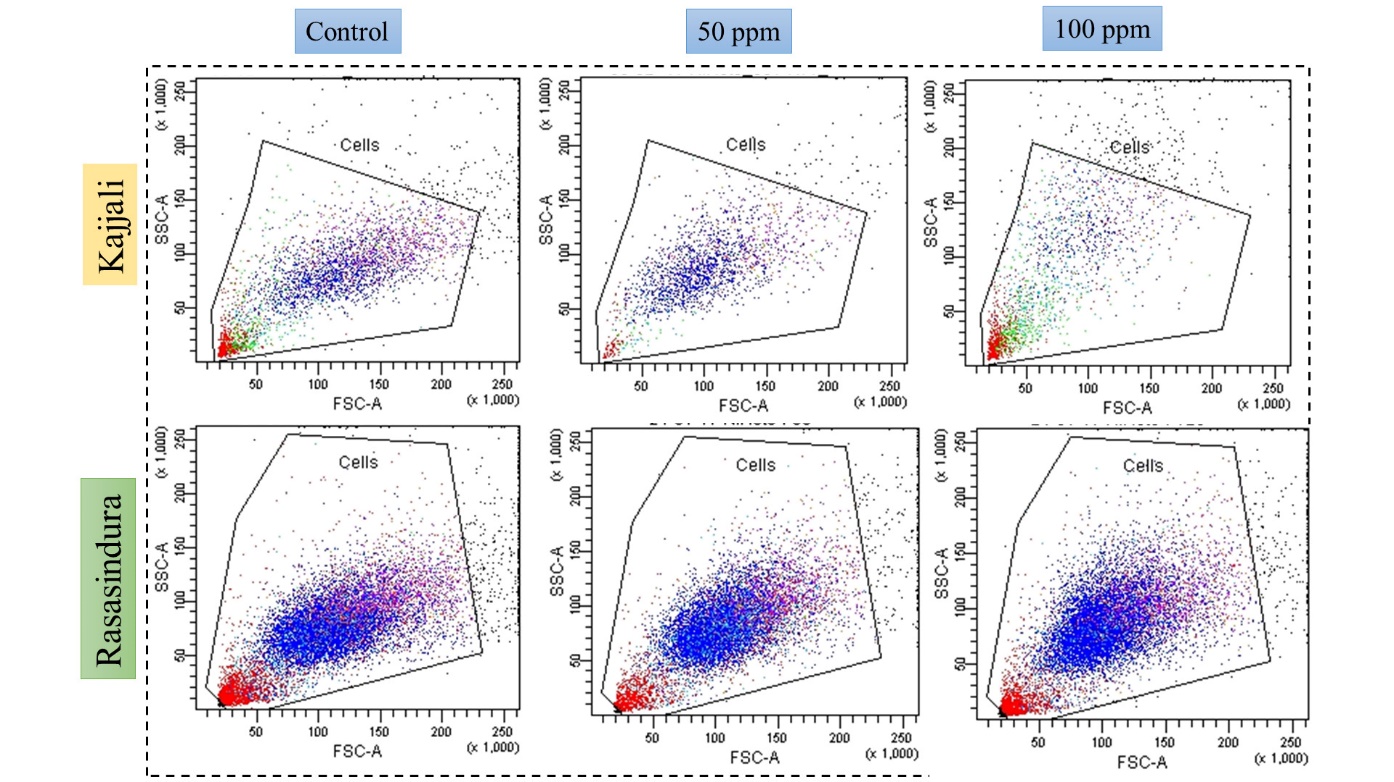


**Figure S5.** FACS study of NIH3T3 cells after 48 hours of *Kajjali* and *Rasasindura* exposure FACS study, SSC v/s FSC curve (48 hours after exposure). As SSC signal increases with increasing concentration of drugs, it can be interpreted that cellular uptake of *Kajjali* and *Rasasindura* particles occur.
